# Virtually-delivered Sudarshan Kriya Yoga (SKY) for Canadian veterans with PTSD: A study protocol for a nation-wide effectiveness and implementation evaluation

**DOI:** 10.1371/journal.pone.0275774

**Published:** 2022-10-26

**Authors:** Justin Ryk, Robert Simpson, Fardous Hosseiny, MaryAnn Notarianni, Martin D. Provencher, Abraham Rudnick, Ross Upshur, Abhimanyu Sud

**Affiliations:** 1 Bridgepoint Collaboratory, Lunenfeld-Tanenbaum Research Institute, Sinai Health System, Toronto, Ontario, Canada; 2 Toronto Rehabilitation Institute, University Hospital Network, Toronto, Ontario, Canada; 3 Temerty Faculty of Medicine, University of Toronto, Toronto, Ontario, Canada; 4 Atlas Institute for Veterans and Families, Ottawa, Ontario, Canada; 5 University of Ottawa Institute of Mental Health Research at The Royal, Ottawa, Ontario, Canada; 6 École de Psychologie, Université Laval, Québec City, Québec, Canada; 7 Centre d’évaluations et d’interventions en santé mentale (CÉISM), Université Laval, Québec City, Québec, Canada; 8 VITAM and CERVO Research Centres, Québec City, Québec, Canada; 9 Departments of Psychiatry and Bioethics and School of Occupational Therapy, Dalhousie University, Halifax, Nova Scotia, Canada; 10 Nova Scotia Operational Stress Injury Clinic, Nova Scotia Health, Dartmouth, Nova Scotia, Canada; 11 Clinical Public Health, Dalla Lana School of Public Health, University of Toronto, Toronto, Ontario, Canada; 12 Department of Family and Community Medicine, Temerty Medicine, University of Toronto, Toronto, Ontario, Canada; 13 Humber River Hospital, Toronto, Ontario, Canada; Kasturba Medical College Mangalore, Manipal Academy of Higher Education, INDIA

## Abstract

**Background:**

Post-traumatic stress disorder (PTSD) remains a significant treatment challenge among Canadian veterans. Currently accessible pharmacological and non-pharmacological interventions for PTSD often do not lead to resolution of PTSD as a categorical diagnosis and have significant non-response rates. Sudarshan Kriya Yoga (SKY), a complementary and integrative health (CIH) intervention, can improve symptoms of PTSD. In response to the COVID-19 pandemic, this intervention has pivoted to virtual delivery and may be reaching new sets of participants who face multiple barriers to care.

**Objective:**

To evaluate the implementation and effectiveness of virtually delivered Sudarshan Kriya Yoga (SKY) on decreasing PTSD symptom severity, symptoms of depression, anxiety, and pain, and improving quality of life in Canadian veterans affected by PTSD.

**Methods and analysis:**

Using a mixed-methods approach guided by the RE-AIM framework, we will conduct a hybrid type II effectiveness and implementation study of virtually delivered Sudarshan Kriya Yoga (SKY) for Canadian veterans. Effectiveness will be evaluated by comparing virtually delivered SKY to a waitlist control in a single-blinded (investigator and data analyst) randomized controlled trial (RCT). Change in PTSD symptoms (PCL-5) is the primary outcome and quality of life (SF-36), symptoms of depression (PHQ-9), anxiety (GAD-7), and pain (BPI) are secondary outcomes. The SKY intervention will be conducted over a 6-week period with assessments at baseline, 6-weeks, 12-weeks, and 30 weeks. The reach, effectiveness, adoption, implementation, and maintenance of the intervention will be evaluated through one-on-one semi-structured interviews with RCT participants, SKY instructors, health professionals, and administrators that work with veterans.

**Discussion:**

This is the first investigation of the virtual delivery of SKY for PTSD in veterans and aims to determine if the intervention is effective and implementable at scale.

## Introduction

Post-traumatic stress disorder (PTSD) was originally identified among soldiers who experienced horrific events on the battlefield [1]. The symptoms of PTSD include, but are not limited to, intrusive recollections including flashbacks, avoidance of reminders of the trauma, alterations in mood or cognition, and hyperarousal [2]. Besides many physical and psychological symptoms associated with PTSD, significant disruption in social and/or occupational functioning can also occur.

PTSD is highly prevalent among Canadian veterans. According to Veterans Affairs Canada (VAC), of the 133,200 veterans receiving benefits from VAC, 24,538 (18.4%) are diagnosed with and receive disability benefits for PTSD. In addition, the prevalence of PTSD among Canadian veterans is rising, indicating that the number of veterans in need of support for PTSD will likely continue to increase over time [3]. Due to various barriers including long wait times, stigma, and uncertain or fragmented financial coverage [4], a significant proportion of veterans may not receive effective treatment for their PTSD.

PTSD is highly comorbid with other psychiatric and physical illnesses, including major depressive disorder (61.5%), generalized anxiety disorder (52.3%), and chronic pain (50%) [5–7]. These comorbidities have significant consequences. Individuals who have both PTSD and depression are more likely to engage in purposeful self-harming behaviors [8]. Additionally, those with comorbid PTSD and social anxiety disorder are more than 3 times more likely than those with PTSD alone to report a past suicide attempt [9].

There are a variety of interventions available for PTSD, with trauma-focused cognitive-behavioral therapies (TF-CBT) demonstrating the largest benefit compared to a control in clinical trials with a standardized mean difference (SMD) of 1.23 (95% CI [0.97, 1.50]) [10–12]. Although trauma-focused psychotherapy has larger effects, pharmacotherapy is also recommended as first-line treatment when trauma-focused psychotherapy is not available or not preferred by the patient [11]. First-line pharmacotherapies include selective serotonin reuptake inhibitors (SSRIs) such as fluoxetine, paroxetine, and sertraline, and the serotonin-norepinephrine reuptake inhibitor (SNRI), venlafaxine [13].

Despite this evidence for efficacy, there remain several treatment challenges with current PTSD therapies. Firstly, treatments often do not lead to resolution of PTSD as a categorical diagnosis and even the most effective trauma-focused therapies have non-response rates as high as 50% [10, 14]. Secondly, pooled data from randomized controlled trials (RCTs) demonstrate that dropout rates are higher for trauma-focused therapies compared to non-trauma-focused therapies, at 18% and 14%, respectively [14, 15]. While the reasons for attrition are not fully known, stigma, confidentiality concerns, significant time demands, therapist fit, and negative expectancy likely play important roles [4]. Thirdly, existing therapies have reduced effectiveness and lower treatment retention in veteran populations compared to civilians [16]. There is thus an evident need for versatile treatments for PTSD and its comorbidities.

The COVID-19 pandemic has exacerbated existing challenges of access to appropriate care [17]. Virtual therapies, in particular telepsychotherapies, have enabled mental health professionals to continue supporting individuals throughout the pandemic. As virtual therapies become more common, research must also focus on implementation in addition to effectiveness to support real-world uptake of interventions. Recent research has shown high acceptance of virtual therapies among veteran populations, while also identifying several barriers, facilitators, and future challenges [18–20]. Barriers can include poor access to high-speed internet, lack of privacy in the home, lack of comfort with technology, and a lack of personal connection with therapists that can be perpetuated with decreased non-verbal cues. Facilitators can include the convenience of using virtual therapies, the comfort of being in one’s home, reduced travel time, and reduced costs due to missed work [19].

In Canada, the use of complementary and integrative health (CIH) interventions has increased steadily over the last several decades; including CIH interventions for PTSD [21]. The rise of CIH interventions among individuals with PTSD may in part be a response to existing challenges of efficacy and accessibility of PTSD treatments [14, 19]. Among veterans in particular, there has been growing interest in CIH interventions [22], with almost 50% of veterans and active duty personnel using at least one CIH therapy [23]. Promising results for meditation and mindfulness-based interventions have been demonstrated for PTSD and many of its comorbidities. Additionally, these interventions have the added benefit of a strong safety profile [24–26].

CIH interventions are complex interventions that involve numerous components such as meditation and breathing exercises, psychoeducation, and group dynamics [27]. Successful implementation of these practices into new contexts and settings necessitates understanding the perspectives of involved stakeholders, including Canadian veterans, instructors, health professionals, and administrators that work with veterans. The availability of resources, administrative and bureaucratic difficulties with integrating CIH interventions in routine care, and balancing patient preferences are among some of the challenges that stakeholders must consider when implementing CIH therapies into veteran health care [28].

Several CIH interventions have demonstrated therapeutic efficacy for improving PTSD symptoms [29–33]. Over the last decade, numerous studies have demonstrated the effectiveness of Sudarshan Kriya Yoga (SKY), a form of breathing-based meditation, for improving PTSD in veteran populations [29, 34–36]. A recently published RCT suggested non-inferiority of SKY compared to cognitive processing therapy for improving PTSD symptoms in US military veterans [37]. Compared to a waitlist control, SKY has shown an effect size of 1.16 for improvement of PTSD symptoms (95% CI [0.20, 2.04]) [29] with retention rates between 81% and 95% [29, 38]. The beneficial effects of SKY are not limited to PTSD, but extend also to major depressive disorder and generalized anxiety disorder [39, 40].

Outside of research settings, SKY is being delivered widely in the United States for combat and non-combat veterans, as well as their families and caregivers. It is taught by highly trained and certified instructors from the International Association for Human Values and Art of Living Foundation. By shifting responsibility for intervention delivery to expert, non-healthcare professionals, SKY may provide a pathway to increase intervention accessibility alongside formal healthcare delivery. SKY is typically delivered to groups of 8–12 people by certified instructors. In response to the pandemic, this intervention has pivoted to virtual delivery. Informal evaluative reports from participants and SKY instructors [personal communication] suggest that virtual delivery creates a similar participant experience of a safe, non-judgemental community of learning, engagement and change. Reports from instructors also suggest that the SKY intervention has reached new sets of participants, including those who suffer from social anxiety or live in rural settings and face geographic barriers to care.

The existing evidence supporting the use of SKY for veterans with PTSD provides the rationale for studying virtually-delivered SKY in Canadian veterans with PTSD. There is a need for high quality-controlled trials in order to generate evidence in a Canadian context for the effectiveness of SKY and to better understand how such an intervention should be implemented.

This protocol article describes the research study “Sudarshan Kriya Yoga (SKY) for Canadian veterans with PTSD: a nation-wide effectiveness and implementation evaluation”. This trial protocol was developed through extensive and iterative consultation with Canadian veterans with lived experience with PTSD, family members of veterans, experts at specialty veteran mental health clinics across Canada, and experts in SKY delivery within the context of research trials. These consultations were conducted to help ensure relevance of the trial to Canadian veterans living with PTSD and feasibility of the trial within the particularities of the contemporary Canadian context. This article describes the procedures, methodology and methods, besides other considerations for a hybrid type II study design of a virtually-delivered meditation intervention.

### Study aim and objectives

The aim of this study is to evaluate the effectiveness and implementation of the virtually-delivered SKY (v-SKY) intervention for Canadian military and RCMP veterans living with PTSD. PTSD symptom severity and several health-related outcomes will be evaluated for effectiveness. The implementation of the v-SKY intervention will also be evaluated by interviewing RCT participants, SKY instructors, health professionals, and administrators that work with veterans. Evaluating implementation of a virtual intervention is relevant in both pandemic and post-pandemic contexts where virtual interventions will likely continue to be widely available and possibly preferred by patients and providers.

The protocol for the effectiveness trial is reported using the Standard Protocol Items: Recommendations for Interventional Trials (SPIRIT) statement [41] and the intervention is described using the Template for Intervention Description and Replication (TIDieR) checklist and guide as well as the CheckList stAndardising the Reporting of Interventions For Yoga (CLARIFY) [42, 43]. The protocol for the implementation evaluation is described according to the Standards for Reporting Implementation Studies (StaRI) statement [44]. A completed SPIRIT table showing the timeline for this study is shown in **Table 1** and a completed SPIRIT Checklist is located in **Section 1 of the S1 Appendix**. The completed World Health Organization Trial Registration Data Set can be found in **Section 2 of the S1 Appendix**.

**Table 1:** Outline of enrolment, study intervention, and effectiveness and implementation evaluations.

Timeline	Pre-study Screening	Screening/Consent	Randomization/Allocation	T1	*Weeks 1–6*	T2	*Weeks 6–12*	T3	T4
				Baseline					
				*Week 0*		*Week 6*		*Week 12*	*Week 30*
**Enrolment**									
Informed Consent Form		X							
Pre-screener script	X								
PCL-5		X					X		
Randomization			X						
MINI		X							
MoCA Mini		X							
**Intervention**									
SKY Intervention–Active (ACT) group					X				
SKY Intervention–wait list control (WLC) group							X		
Attendance logs					X		X		
**Assessments**									
Intake questionnaire				X					
PCL-5				X		X		X	X
CAPS-5				X					
PHQ-9				X		X		X	X
GAD-7				X		X		X	X
BPI				X		X		X	X
SF-36				X		X		X	X
Medication History				X		X		X	X
**Implementation Evaluation**									
Post-intervention questionnaire						X (ACT group)		X (WLC group)	
**Timeline**	After T3 assessment			After completion of last cohort			Post study		
Qualitative interviews–RCT participants	Participants will be identified		X	X					
Qualitative interviews–Stakeholder participants	Participants will be identified					X	X		
Qualitative interviews–Instructor participants	Participants will be identified					X	X		

Abbreviations: PTSD Checklist for DSM-5 (PCL-5), Mini International Neuropsychiatric Interview (MINI), Montreal Cognitive Assessment mini version (MoCA Mini), Clinician Administered PTSD Symptom Scale (CAPS-5), Patient Health Questionnaire-9 (PHQ-9), Generalized Anxiety Disorder Scale (GAD-7), Brief Pain Inventory (BPI), Health-Related Quality of Life measurement (SF-36)

#### Primary objectives

*Effectiveness evaluation*. In Canadian veterans with clinically significant PTSD symptoms, we will estimate the short-term (6-weeks) effectiveness of v-SKY compared to a waitlist control at reducing PTSD symptoms as measured by PTSD Checklist for DSM-5 (PCL-5).

*Implementation evaluation*. We will explore adoption of v-SKY by examining the intervention’s acceptability, accessibility, and perceived effects amongst Canadian veterans with PTSD and amongst health professionals, and administrators that work with Canadian veterans.

#### Exploratory objectives

We will estimate the short- and long-term effects of v-SKY on quality of life, depression, anxiety, and chronic pain in Canadian veterans affected by PTSD. In addition, we will evaluate the reach, adoption, implementation, and maintenance of the v-SKY intervention amongst Canadian veterans with PTSD, SKY instructors, and the health professionals, and administrators that work with veterans.

## Materials and methods

### Overall research design

We will conduct a hybrid type II effectiveness and implementation evaluation guided by the RE-AIM framework [45]. RE-AIM provides a framework for measuring the success of translating findings from effectiveness research into practice by evaluating *effectiveness* along with the *reach*, *adoption*, *implementation*, and *maintenance* of an intervention on an individual and organizational level. The RE-AIM framework facilitates the study of an intervention under more complex, real-world conditions as opposed to optimized and controlled conditions typical of trials aiming to maximize internal validity [45].

### Trial design

**Fig 1:**
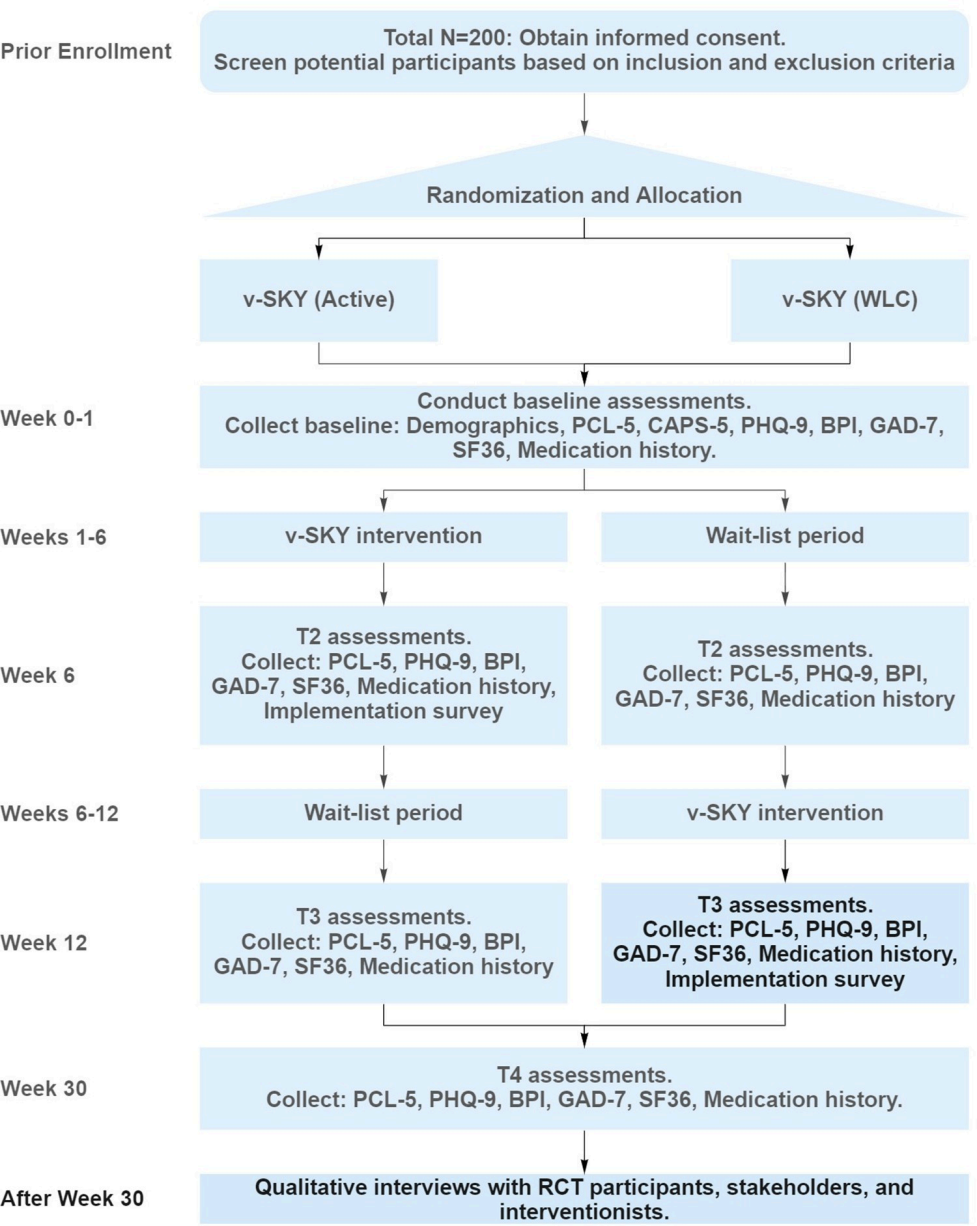
Flowchart showing the timeline of the randomized controlled trial.

Effectiveness will be evaluated with a randomized, waitlist controlled, single-blinded (investigator and data analyst), cross-country trial conducted remotely using video calls with a primary endpoint of changes in PTSD symptom severity. Randomization will be performed with a 1:1 allocation to either the active intervention or waitlist control group. This study will be conducted out of the Sinai Health System together with the Atlas Institute for Veterans and Families, with study promotion and recruitment support from several clinical and community-based organizations across the country. Evaluations of PTSD symptom severity, depression, anxiety, pain, and quality of life will be administered at baseline, 6-weeks, 12-weeks, and 30-weeks follow-ups (**Fig 1)**.

This study was originally conceived as a comparative effectiveness study using a non-inferiority approach, similar to the US study described by Mathersul et al [37, 46]. This proposed trial would have compared virtually-delivered SKY (v-SKY) to virtually-delivered cognitive processing therapy (CPT) in a non-inferiority trial, with the key innovation being virtual delivery of SKY. However, formal consultation with experts in veteran mental health care and veterans who have lived experience with PTSD indicated that the SKY intervention would likely be most useful in a Canadian context if it were to be integrated into the current treatment environment instead of being compared to a standard of care treatment. For instance, many patients in specialty clinics have not responded to existing treatments, which emphasized a need for more complementary therapeutic options. These discussions also identified wait times for access to care between 4–8 weeks, which is comparable to the wait list control time period. Likewise, as per our earlier estimates, a high number of veterans with PTSD may not be actively receiving treatment for their illness. Thus, a wait list control design was considered as the most appropriate, relevant, and feasible.

Several RCTs of SKY for veterans living with PTSD have been conducted using waitlist control designs including in the United States [29] and Australia [38]. Using a waitlist control design encompasses using a control group that is not actively receiving treatment for their illness during the intervention period. Baseline assessments will be conducted with the active intervention (ACT) and waitlist control (WLC) groups before the active intervention group receives the intervention. After the 6-week intervention period for the ACT group, there will be a 2-week data collection period for both the ACT and WLC groups. After this 2-week period, the WLC group will begin the intervention for 6-weeks and the ACT group will not. After the WLC group’s 6-week intervention, there will also be a 2-week data collection period for both groups.

#### Study population and recruitment

For the RCT, 200 participants who are veterans of the Canadian Armed Forces (CAF) or Royal Canadian Mounted Police (RCMP) will be recruited through various organizations and clinics across Canada. Military and RCMP veterans are groups of interest for this study because they are the specific recipients for health benefits from VAC and are the population of focus for the Atlas Institute for Veterans and Families. A list of organizations and clinics that work with Canadian veterans that are supporting recruitment are provided in **Section 3 of the S1 Appendix**. Screening and study assessments will be conducted virtually out of Sinai Health System in Toronto, Ontario.

Study promotional materials, which include a poster, brochure, and website will be used for recruitment. These materials will be distributed by the organizations and clinics supporting recruitment. Interested participants can self-refer by completing a pre-screener survey that is linked through the study website, which allows them to leave their contact information if they may be eligible. Participants can also directly reach out to the study staff via phone and e-mail information on the promotional materials. Specialized clinics that work with Canadian veterans will also identify eligible participants and refer them directly to the study staff for further screening. Study materials will also be prepared in French for the recruitment of Francophone participants.

#### Screening

A virtual screening visit will involve fully informing potential participants about the study, providing them an opportunity to ask questions, and then obtaining informed verbal consent from those that are interested in participating. For those who consent, there will be a subsequent screening for inclusion and exclusion criteria (shown in **Table 2**), which includes administration of the Mini Neuropsychiatric Interview (MINI), the PCL-5, and the Montreal Cognitive Assessment (MoCA) mini version.

**Table 2:** Outline of inclusion and exclusion criteria and their justifications.

Criteria	Rationale
**Inclusion Criteria**	
Aged 18 years or older.	The population of interest, Canadian veterans with PTSD, are all adults.
CAF or RCMP veteran.	Military and RCMP veterans are a population that experiences PTSD at rates higher than a civilian population. Furthermore, a significant number of veterans are not connected with care through VAC [3]. SKY has been studied in veteran populations, making Canadian veterans a good target population for this study.
Meet criteria for current PTSD as determined by a score of >38 on the PTSD Checklist-5 (PCL-5) [47].	The PCL-5 is a well-validated, 20-item self-report measure for screening and assessing current PTSD symptom severity according to the Diagnostic and Statistical Manual of Mental Disorders (DSM-5) [2, 47]. A score of 31–33 is a cut-off score that has the highest convergent validity with CAPS-5, the gold standard for assessing PTSD symptom severity [48]. The score of 38 is chosen as a conservative estimate of clinically meaningful symptoms of PTSD and has also been used in a previous clinical trial [37, 46].
**Exclusion Criteria**	
Participation in another concurrent clinical trial.	To limit confounding effects of other trials.
Intention to begin a new course of Behavioral Therapy (e.g., Cognitive Behavioral Therapy including Prolonged Exposure or Cognitive Processing Therapy) during the intervention period.	To limit confounding effects from other treatments during the 6-week intervention period. Participants may be eligible 8-weeks after they complete a formal behavioral therapy program.
Past participation in virtual or in-person SKY.	To limit the study intervention as a confounding variable.
Initiated psychotropic medication within 8 weeks prior to the initial screening. Excluded participants could be re-considered for eligibility after stability on medication was achieved.	To limit confounding effects from pharmacological therapies.
Risk of imminent suicidal intent at screening as per MINI screening and standard tool. Such patients will be advised to visit their local emergency department.	Effects of SKY on more severe health conditions are unknown.
Mental disorders including schizophrenia, bipolar I, psychosis of any type.	
Seizure disorder not well controlled.	
Moderate or severe substance use disorder as per the MINI.	
Neurocognitive impairment as determined by a score of 23 or less on the Montreal Cognitive Assessment (MOCA) mini version [49].	Those with any cognitive impairment may have difficulties fully participating in v-SKY sessions.
Unable to connect to the internet and use Zoom for study assessments and interventions.	Because these interventions will be virtual, it is important for participants to have a stable internet connection and a device with a camera and microphone that supports video calls.
Inability to provide informed consent.	As per ethical norms for research involving human subjects, people who cannot provide informed consent due to capacity/competency reasons, medical reasons, literacy or language limitations, etc. will not be included in the trial.

#### Consent

Research staff will first obtain verbal consent from interested participants. We will obtain formal, signed consent from participants who are screened eligible. Those who are able to provide a signed copy of the consent form will be asked to return it by email; a copy of the completed documentation of verbal consent will be sent via email as an attachment. For participants that are unable to provide a signature (e.g. due to lack of a printer and scanner, or software to add an electronic signature), documentation of verbal consent will serve as the record of obtained consent. Participants may consent to the use of their data for future studies and analyses.

#### Institutional approval and registration

This study has received approval by the Mount Sinai Hospital Research Ethics Board (REB approval number: 21-0275-A) and has been registered on clinicaltrials.gov (NCT05235828).

#### Randomization and allocation

Participants will undergo 1:1 randomization and allocation to the active v-SKY intervention group or a waitlist control (WLC) group using the data management and randomization services available at Sinai Health System. This will be used as a central repository for the generation of randomization codes and will ensure concealment of randomization. A member of the research team not involved in recruitment or evaluations will contact participants with randomization and allocation information to maintain blinding. Participants will be allocated into either the active or WLC arms of cohorts that will be running on a rolling basis. Participants randomized to the active arm will receive the v-SKY intervention in groups of 8–12. Participants allocated to the WLC arm will receive the v-SKY intervention after a waitlist period of approximately 6 weeks. It will not be possible for participants to be blinded to their intervention status, but they will be instructed to not share allocation information with study staff. Separate groups for Francophone speakers will be delivered in French on a rolling basis.

*Trial intervention*. SKY meditation is a standardized intervention that includes relaxation techniques as well as periods of group discussion [34, 46]. This practice incorporates different breathing techniques centered around controlling arousal and attention. Initially, the breathing techniques that are taught are calming and focusing. While progressing through the sessions, breathing exercises are more engaging and energizing, which allows the practitioner to focus more in each moment. Participants will be encouraged to learn all of the techniques that are covered during these sessions and also utilize the techniques that are most appropriate for their personal needs. After some initial training sessions, participants will be encouraged to practice their new SKY techniques in environments and situations that they have been avoiding. They would begin with situations that are less triggering, and progress to more difficult situations. At each group session, participants will describe their experiences and discuss ways to continue to incorporate the SKY techniques into their daily lives, until it reaches a point of being natural to use these skills throughout the day, both in practice, and in application.

Experienced and licensed SKY instructors from the International Association for Human Values and Art of Living Foundation will provide the intervention using the video-calling software, Zoom. Instructors undergo a two-year pre-training program followed by a 16-day residential training workshop, a practicum, and subsequent mentorship program. Upon completion of the mentorship program, instructors can facilitate the SKY intervention on their own and are considered fully certified. Instructors providing SKY training for veterans go through an additional 100 hours of specialized training to meet the specific needs of veterans.

Similar to other studies of SKY for veterans living with PTSD [46], instructors will guide participants through the v-SKY intervention through visual demonstrations and verbal guidance. For the first 5 days, groups will meet for 3-hour sessions, followed by five weeks of 1-hour sessions twice per week (**Table 3**). In total, the intervention will be 15 sessions that run for a total of 25 hours. Sessions will not be recorded.

**Table 3:** A description of the 6-week Sudarshan Kriya Yoga intervention.

Week 1	Sessions 1–5	Participants will be introduced to SKY meditation and breathing practices. Some of these practices and techniques that are covered include ujjayi or “Victorious Breath,” bhastrika or “Bellows Breath,” “om” meditation chants, and the sudarshan kriya, which involves advanced forms of cyclical breathing. Participants will be assigned homework to practice SKY breathing practices for 5, 10, 15, 20, and then 30 minutes. After these first 5 sessions, participants will be asked to practice SKY breathwork for 30 minutes daily.
Weeks 2–6	Sessions 6, 8, 10, 12, 14	Participants will learn more SKY breathing and accompanying practices.
	Sessions 7, 9, 11, 13, 15	Participants will learn guided meditation and home practice programs to facilitate the continuing practice of SKY.

Attendance and homework logs will be used to monitor adherence to the SKY protocol for each cohort.

#### Outcome measures and assessments

For the effectiveness trial, change in PTSD symptoms is the primary outcome measure (**Table 4**). Changes in depressive, pain, anxiety symptoms and quality of life are exploratory outcome measures.

**Table 4:** Primary and exploratory outcome measures for the effectiveness evaluation.

Outcome Measure	Explanation
Change in PTSD symptoms *Primary*: Change in PCL-5 scores from baseline to post-treatment (6-weeks).	The PCL-5 is a well-validated, widely used, and easy to administer 20-item self-report scale that is widely used in PTSD research and clinical practice [47]. Responses to each item relate to the four symptom clusters for PTSD, for which separate scores can be measured and used for additional analyses. The PCL-5 will be used for follow-up assessments over the CAPS-5 because of the feasibility of a self-report measure over a semi-structured interview and to reduce the reporting burden on participants.
*Exploratory*: Prolonged changes in PCL-5 scores from baseline to 12-week follow-up and 30-week follow-up.	
Change in depressive symptoms *Exploratory*: Change in depressive symptoms as measured by the nine-item Patient Health Questionnaire (PHQ-9) scores from baseline to 6-weeks, 12-weeks, and 30-weeks follow-ups.	PHQ-9 is a well-validated and widely used self-report scale used in depression research. Each item is rated on a 4-point scale for how much it bothered the participant in the past 2 weeks. A self-report scale is more feasible than an assessor-rated scale (e.g. HAM-D17) given the virtual nature of this study.
Change in pain symptoms *Exploratory*: Change in pain symptoms as measured by the Brief Pain Inventory (BPI) from baseline to 6-weeks, 12-weeks, and 30-weeks follow-ups.	BPI is a validated self-report scale used in pain trials and clinical pain practice and is a core outcome measure per the Initiative on Methods, Measurement, and Pain Assessment in Clinical Trials (IMMPACT) recommendations [50]. Two independent and clinically relevant measures are included: pain severity and pain interference with function.
Change in anxiety symptoms *Exploratory*: Change in anxiety symptoms as measured by the seven-item Generalized Anxiety Disorder scale (GAD-7) from baseline to 6-weeks, 12-weeks, and 30-weeks follow-ups.	GAD-7 is a validated and efficient tool that is used to screen and assess the severity of generalized anxiety disorder GAD clinically and in research. Each of the 7-items are rated on a 4-point scale for how often it bothered the participant in the past two weeks.
Change in Quality of Life *Exploratory*: Change in quality of life as measured by the 36-item Health-Related Quality of Life measurement (SF-36) from baseline to 6-weeks, 12-weeks, and 30-weeks follow-ups.	Change in health-related quality of life (SF-36) has been validated as quality of life measurement and is recommended by IMMPACT [50]. This measure is well-aligned to the intervention which may also improve overall quality of life (QoL) in addition to specific PTSD symptom improvement.

In addition to these primary and exploratory outcome measures, participants will also complete an intake survey for their baseline assessment. This survey will collect demographic information on age, partial date of birth (DOB), gender, race, ethnicity, employment status, military and/or RCMP service status, housing type, highest level of education achieved, smoking, caffeine intake, drug and alcohol use, duration of PTSD diagnosis, and past treatment history for their PTSD. Participants will also be asked about their psychiatric history including age of first contact with services for mental illness (and which illness(es)), history of hospital admissions, and health service use. We will also collect data on medications used by participants including names, dosages (per day, total daily), and the reason for their use. This will include vitamins/minerals/herbal preparations and other over-the-counter (OTC) medications. Participants will not be excluded from the study for medication changes. Any changes to medications will be captured at follow-up assessments.

At the follow-up assessment timepoints, the following questionnaires will be administered: PCL-5, PHQ-9, BPI, GAD-7, SF-36. In addition, a post-intervention questionnaire will be administered after the v-SKY intervention. This will be at the T2 assessment for the active intervention group and the T3 assessment for the WLC group. This questionnaire will collect information on barriers and facilitators for the v-SKY intervention, the intervention’s instruction and setting, and level of satisfaction with the intervention. A flowchart of the study design is shown in **Fig 1**.

### Implementation evaluation design

The implementation evaluation will be conducted in parallel to the effectiveness trial based on the experiences and perspectives of RCT participants, v-SKY instructors, as well as health professionals and administrators that work with veterans. For RCT participants, this implementation evaluation will be conducted with participants who pass the T3 assessment timepoint. For health professionals and administrators and instructors, the evaluation will be conducted at the end of the effectiveness trial.

#### RE-AIM framework

The five dimensions of the RE-AIM framework will be explored amongst Canadian veterans with PTSD, v-SKY instructors, health professionals, and administrators that work with Canadian veterans (**Table 5**). We are focusing on adoption from the perspective of Canadian veterans and the people who work with Canadian veterans for this study because v-SKY is in the early stages of its implementation.

**Table 5:** Descriptions of the methodological approaches used to assess each RE-AIM dimension in each population.

RE-AIM dimension	Veterans	SKY Instructors	Health professionals and administrators
**Reach**	*Quantitative approach*: Measuring recruitment metrics, attrition, and demographics using detailed tracking logs and questionnaires. Comparison of baseline demographics to observational studies of Canadian veterans with PTSD [5–7] to determine representativeness.	Not assessed amongst instructors.	*Qualitative approach*: Assessing interest in the research study and for one-on-one semi-structured interviews.
**Effectiveness**	*Mixed-methods approach*: Measuring effects of intervention on health-related outcomes using validated questionnaires and assessing indicators of effectiveness through semi-structured interviews.	*Qualitative approach*: Assessing indicators of the v-SKY intervention’s effectiveness through semi-structured interviews.	*Qualitative approach*: Assessing indicators of the v-SKY intervention’s effectiveness through semi-structured interviews.
**Adoption**	*Mixed-methods approach*: Measuring the frequency of which SKY techniques are used and the factors that contribute to continued use of this practice using the combination of a post-intervention questionnaire and semi-structured interviews.	*Qualitative approach*: Assessing factors that may contribute to adoption of SKY techniques by participants through semi-structured interviews.	*Qualitative approach*: Assessing factors that contribute to adoption of the v-SKY intervention through semi-structured interviews.
**Implementation**	*Qualitative approach*: Assessing barriers and facilitators to implementation of the v-SKY intervention using the combination of a post-intervention questionnaire and semi-structured interviews.	*Mixed-methods approach*: Assessing barriers and facilitators to implementation of the v-SKY intervention through semi-structured interviews and cohort completion questionnaires.	Not assessed amongst health professionals and administrators.
**Maintenance**	*Qualitative approach*: Assessing whether or not participants are continuing to practice SKY and what factors contribute to this continued use through semi-structured interviews.	Not assessed amongst instructors.	Not assessed amongst health professionals and administrators.

#### Conceptual framework for implementation evaluation

While RE-AIM is used as the conceptual framework for studying effectiveness and implementation in this hybrid type II study, we will also employ Normalization Process Theory (NPT) as a means to understand adoption within the RE-AIM framework. NPT aims to estimate how likely an intervention may be incorporated into routine practice by highlighting the various facilitators and barriers in the early stages of its implementation [51]. NPT helps delineate how stakeholders make sense of an intervention, the cognitive and relational work required to participate, the collective actions required to make the intervention work and how to appraise outcomes and ongoing use (**Table 6**). NPT has been used elsewhere to evaluate implementation of mind-body interventions for individuals with multiple sclerosis [52].

**Table 6:** A Description of each core construct of Normalization Process Theory.

NPT Construct	Description
Coherence	Sense-making work for operationalizing a new program.
Cognitive Participation	Relational work done to sustain a community of practice around a new program.
Reflexive Monitoring	An ongoing assessment/appraisal of how new programs affect various stakeholders and the individuals around them.
Collective Action	The operational work conducted by stakeholders in order to enact a new program.

To integrate and implement the v-SKY intervention for Canadian veterans with PTSD, it is necessary to consider how the intervention is likely to be understood by the relevant stakeholders. Firstly, the SKY intervention is relatively new to Canadian veterans and the organizations and clinics that work with them. Although the intervention has been studied in other veteran populations, it is important to understand implementation in the Canadian context. Secondly, this is the first study focusing on the virtual implementation of the SKY intervention. It is important to study how virtual programming fits within the current treatment environment.

NPT has four main constructs that represent the different types of work that people do when faced with a new intervention such as v-SKY (**Table 6**). Adoption will be evaluated using each of these four constructs, which will be explored through one-on-one semi-structured interviews with RCT participants, SKY instructors, health professionals, and administrators that work with Canadian veterans.

#### Study population

The study populations for the implementation evaluation will consist of the veteran RCT participants recruited from across Canada, SKY instructors from the International Association for Human Values and Art of Living Foundation, and health professionals and administrators that work with veterans.

#### Participant recruitment and consent

*RCT participants*. Up to 16 RCT participants will be recruited for one-on-one semi-structured interviews. Participants who completed the v-SKY intervention and those who did not complete or dropped out of the intervention will be identified using criterion sampling. This will ensure that the perspectives of people who did not participate in the full trial will be assessed. Both completers and non-completers will be recruited for these interviews. Participants will begin to be identified for interviews after the T3 assessment timepoint, which is when the completion or non-completion status will be known. For these interviews, we aim to include French-speaking participants proportionate to the overall trial recruitment. An interview guide is located in **Section 4a of the S1 Appendix.**

*SKY instructors*. The SKY instructors from the International Association for Human Values and Art of Living Foundation that led v-SKY sessions throughout this study will be identified for one-on-one semi-structured interviews to discuss the virtual implementation of the v-SKY intervention. Informed consent will be obtained by research staff prior to any formal assessments. Convenience sampling will be used to recruit up to 8 interested instructors for these interviews. These instructors will provide further information about the virtual implementation of the v-SKY intervention by reflecting on their experiences in the trial. Recruitment of instructors will take place after the last cohort in the RCT trial is complete. An interview guide is located in **Section 4b of the S1 Appendix.**

Instructors will also complete a questionnaire after the completion of each cohort. The cohort completion questionnaire will allow instructors to document any relevant changes or deviations to the protocol within a cohort. This data will be used to understand the virtual implementation of SKY.

*Health professionals and administrators*. Health professionals and administrators that work with veterans will be identified by the study staff, investigators, and/or collaborators over the course of the study. Informed consent will be obtained by research staff prior to any formal assessments. Participants will include clinicians, clinic staff, and policy makers in relevant organizations pertaining to the care of people with PTSD. Stratified purposeful sampling will be used to identify candidates who are interested and able to participate in one-on-one interviews. Up to 16 stakeholders in leadership positions at OSI clinics and organizations that work with Canadian veterans will be recruited. Half of the participants will consist of clinicians who work with veterans, while the other half will consist of managers and personnel in leadership positions at clinics and organizations that work with veterans. Clinicians will be selected for their perspectives on implementation of the v-SKY intervention using their clinical expertise. Those in management positions will be selected for their perspectives on adoption of the v-SKY intervention using their knowledge of organizational and structural barriers. Recruitment of stakeholder participants will take place after the last cohort in the RCT trial is complete. For these interviews, we aim to include French-speaking personnel proportionate to the overall trial recruitment. An interview guide is located in **Section 4c of the S1 Appendix.**

We expect to identify emerging themes within the first six interviews with RCT participants, SKY instructors, and health professionals and administrators [53, 54]. Interviews will continue until data saturation occurs [53].

### Data management and confidentiality

All questionnaires and forms will be completed digitally using surveys hosted through Qualtrics and stored on a secure server based at Sinai Health System. All data will be coded with unique participant IDs to protect patient confidentiality and documents linking participants to their IDs will be stored on a secure database at Sinai Health System and maintained by the research staff for the duration of the study. The code key with identifying data will be stored on a secure server and will only be accessible to the research staff for the duration of the study, unless extenuating circumstances where a participant’s health is at risk and requires unblinding. The research team at Sinai Health System will also be in charge of maintaining the trial protocol and updating it with any amendments as approved by the relevant research ethics boards. They will update participants with any relevant modifications to the protocol and update the trial registry at clinicaltrials.gov accordingly.

RCT participants will be offered the v-SKY intervention at no financial cost. Participants are not expected to incur any additional financial costs to participate in this intervention and the associated assessments. RCT participants that complete all their assessments (up to and including the T4 assessment) will be entered into a draw for gift cards ($25 value). Participants will have a 10% chance of receiving a gift card for completing all study assessments. Any identifying information that is required for sending gift cards or conducting the draw will be stored separately from study information to prevent reidentification of the participant.

Participants will be informed that their participation in this study is voluntary. They may decide not to be in this study, or to be in the study now and then change their mind later. Leaving the study at any time will not affect their care at the partnering clinics and organizations. Participants may also refuse to answer any question they do not want to answer, or not answer an interview question by saying “pass”. If participants withdraw from the study early, any information that was collected will still be used for the study unless they request otherwise. No new information will be collected from them.

### Risks and safety

Previous investigations of SKY have found that SKY is well tolerated among participants with no reported adverse events [29, 55, 56]. Some theoretical adverse effects that could be experienced with SKY are mild anxiety, mild boredom, or mild detachment from one’s surroundings. Participants experiencing any new adverse effects will be advised to consult with their primary care provider. Instructors will be able to connect with study physicians if there are any acute medical issues during the v-SKY intervention. Instructors will consult with study staff to determine if participants experiencing such adverse effects should be permitted to continue. If considered medically unsafe to continue, the participant will be removed from the study and will be advised to consult with their primary health care provider or local emergency department. Participants will be assessed for suicidal ideation during each assessment (positive response to PHQ-9 question 9 ≥1). Any indication of a change in the participant’s overall mental health or risk of suicide will be reported immediately to study physicians for appropriate management. The questionnaires used in the study carry little risk, but participants may feel uncomfortable, sad, embarrassed, or anxious as some of the questions involve thinking about past trauma. Participants may refuse to answer questions or stop the interview at any time if there is any discomfort. There are no known nor expected risks for instructors, health professionals, and administrators that work with veterans.

### Trial advisory committee

An advisory committee has been formed to support this project. The role of the advisory committee will be to: 1) monitor and supervise progress of the trial; 2) support recruitment by engaging with clients and patients through their networks; 3) review, at regular intervals, relevant information about the study; 4) provide feedback to consider how study findings can be presented and used in the future; 5) inform the development and execution of an integrated knowledge mobilization plan.

Due to the low risk of the trial intervention, there will not be a formal data safety and monitoring board, as approved by the governing research ethics board.

## Data analysis

### Sample size justification

The primary outcome is a continuous quantity measured using the PCL-5. The sample size is calculated to have 80% power at 5% type I error. Data from a previous waitlist control study of SKY gives us data to calculate an appropriate sample size. A study by Seppälä et al. used the PCL-M (a DSM-IV version of the PCL-5) for their primary outcome measures [29]. With a conservative standard deviation of magnitude 11 and an effect size of 5, 76 participants must be recruited in each group. A conservative dropout rate of 25% is projected for this study, which has been observed in other clinical trials for PTSD [4, 57]. Accounting for this expected dropout rate, 200 participants will be recruited, with 100 in each group.

### Effectiveness

The primary outcome (PCL-5) will be measured at baseline, 6-weeks, 12-weeks and 30 weeks. The distribution of PCL-5 scores will be examined and an appropriate transformation to correct the possible non-normality will be applied if required. Repeated measures analysis of variance will be used to compare the active intervention and WLC groups at T2, T3, and T4 while adjusting for baseline measures. The demographic characteristics, including age, sex, gender, and baseline symptom severity will be compared between intervention and control groups and the comparison of the primary outcome will be adjusted for significant confounders that may be identified over the course of the study. If significant, this will be included in the repeated measures analysis of variance for adjustment. Descriptive and exploratory statistics will be used to describe and compare the exploratory outcomes (depression, anxiety, pain, and quality of life) between intervention and control groups [58]. This will be an intention-to-treat analysis, where all participants who are randomized will be included in the analysis. There are no pre-planned subgroup analyses.

### Implementation

Data collected through the post-intervention survey will first be analyzed using descriptive statistics. Data collected from demographic questionnaires and recruitment trackers will help describe the reach of the intervention. Baseline demographic characteristics will be compared to observational studies of Canadian veterans with PTSD [5–7] to determine representativeness of the recruited participants.

Data collected through one-on-one semi-structured interviews will be first analyzed thematically using an inductive approach to identify and report themes in the experiences and preferences amongst participants [59]. Data collected from interviews will be uploaded into NVivo to be coded and categorized. The transcripts will then be analyzed thematically using a deductive approach under the core constructs of the NPT framework [51]. Interviews will be pre-categorized as described in the “Participant recruitment and consent” sub-section of the Implementation Evaluation section (e.g. interviews with clinicians, interviews with non-completers of the v-SKY intervention, etc.) and codes will be identified under these interview categories. The inductive thematic analysis is important for limiting potential for researcher bias, which can be greater when conducting a deductive analysis alone. To provide for multiple perspectives on the meaning of the data and how to categorize predetermined themes under the NPT framework, several individuals will read and interpret the transcripts from the interviews. Two members of the research team will review the coded data separately to identify concepts, key words and reflections, and then compare their results with the other member. Initial themes will be reviewed with a limited number of participants to improve data trustworthiness (i.e. ‘member checking’). Throughout analysis, if new themes continue to be identified, we will continue to conduct additional interviews until thematic saturation is achieved.

Investigators involved in the study have previous experience in both inductive and deductive thematic analysis and NPT and, working reflexively, will constantly seek to ensure that findings remain traceable to original participant words/data at all times. All study investigators will have access to the final dataset.

## Discussion

This project will provide much-needed further evidence for interventions for improving PTSD for military and RCMP veterans. Additionally, this study is the first investigation of SKY being delivered virtually for improving PTSD and can provide further evidence around the implementation of virtually-delivered interventions in general. By assessing implementation amongst relevant stakeholders, the intervention’s effectiveness, scalability, and implementability can be evaluated. Therefore, if v-SKY is found to be effective in this population, this could lead to scalable improvements in health service delivery in veteran populations as well as other similar groups with PTSD.

As outlined above, many veterans do not access, or are unable to access, appropriate treatment for PTSD. For such individuals, v-SKY may also be better than no treatment, while having the benefits of being safe and accessible. Our evaluation of the virtual implementation of this intervention may provide valuable information on reaching veterans living in rural communities that are affected by geographic barriers.

This study has some limitations. There is no evidence of SKY leading to a resolution of PTSD as a categorical diagnosis—benefits of the intervention are expected to be more moderate. There is also a potential for selection bias due to the voluntary nature of recruitment. We are recruiting participants from multiple community organizations and specialty clinics to mitigate this bias. We will assess for the representativeness of our sample by comparing to recent observational data about our population of interest. Additionally, this study is limited to those who have access to internet and video-calling technology. Because much of the trial recruitment will centre around larger cities, veterans living in rural areas, who already have limitations in access to care, might be under-represented. Though the waitlist control design was designed to be better reflective of the contemporary knowledge needs in the Canadian context, it does not allow for direct comparison of v-SKY to other PTSD interventions. While we acknowledge these limitations, there are several future applications for this research. Studying implementation provides the groundwork for implementing the intervention more broadly across Canada. Due to the lower upfront costs of v-SKY and its prior efficacy compared to standard trauma-focused therapies, v-SKY holds potential as a cost-effective intervention for PTSD. There may also be a benefit to the healthcare system because v-SKY may reduce healthcare utilization among veterans with PTSD. This is plausible because v-SKY teaches techniques that can be practiced independently by veterans after the intervention is completed. Future studies may also focus on economic analyses of this intervention for this population.

## Supporting information

S1 Appendix (DOCX)
