# Supplementary material for: Virtually-delivered Sudarshan Kriya Yoga (SKY) for Canadian veterans with PTSD: A study protocol for a nation-wide effectiveness and implementation evaluation
Source: PLoS One. 2022 Oct 26;17(10):e0275774. doi: 10.1371/journal.pone.0275774 (PMC9605019; doi:10.1371/journal.pone.0275774)
Supplement: S1 Appendix — (DOCX) [file pone.0275774.s001.docx]

**Supplementary Appendix**

**S1 Appendix: Completed SPIRIT checklist**

**
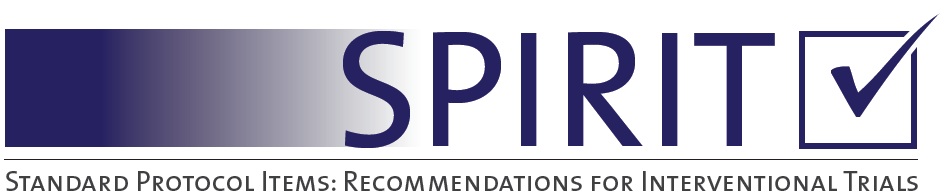
**

SPIRIT 2013 Checklist: Recommended items to address in a clinical trial protocol and related documents*

| **Section/item** | **Item No** | **Description** | **Addressed on page number/section** |
| --- | --- | --- | --- |
| **Administrative information** | | |  |
| Title | 1 | Descriptive title identifying the study design, population, interventions, and, if applicable, trial acronym | 1 |
| Trial registration | 2a | Trial identifier and registry name. If not yet registered, name of intended registry | 2 |
|  | 2b | All items from the World Health Organization Trial Registration Data Set | Supplementary Appendix B |
| Protocol version | 3 | Date and version identifier | Version 1.3 |
| Funding | 4 | Sources and types of financial, material, and other support | Financial disclosure section |
| Roles and responsibilities | 5a | Names, affiliations, and roles of protocol contributors | 1, 2 |
|  | 5b | Name and contact information for the trial sponsor | Financial disclosure section |
|  | 5c | Role of study sponsor and funders, if any, in study design; collection, management, analysis, and interpretation of data; writing of the report; and the decision to submit the report for publication, including whether they will have ultimate authority over any of these activities | Financial disclosure section |
|  | 5d | Composition, roles, and responsibilities of the coordinating centre, steering committee, endpoint adjudication committee, data management team, and other individuals or groups overseeing the trial, if applicable (see Item 21a for data monitoring committee) | 25 |
| **Introduction** |  |  |  |
| Background and rationale | 6a | Description of research question and justification for undertaking the trial, including summary of relevant studies (published and unpublished) examining benefits and harms for each intervention | 4-6 |
|  | 6b | Explanation for choice of comparators | 7 |
| Objectives | 7 | Specific objectives or hypotheses | 8-10 |
| Trial design | 8 | Description of trial design including type of trial (eg, parallel group, crossover, factorial, single group), allocation ratio, and framework (eg, superiority, equivalence, noninferiority, exploratory) | 10-11 |
| **Methods: Participants, interventions, and outcomes** | | |  |
| Study setting | 9 | Description of study settings (eg, community clinic, academic hospital) and list of countries where data will be collected. Reference to where list of study sites can be obtained | 11-12 and Supplementary Appendix C |
| Eligibility criteria | 10 | Inclusion and exclusion criteria for participants. If applicable, eligibility criteria for study centres and individuals who will perform the interventions (eg, surgeons, psychotherapists) | 12-13 (Table 2) |
| Interventions | 11a | Interventions for each group with sufficient detail to allow replication, including how and when they will be administered | 14-16 |
|  | 11b | Criteria for discontinuing or modifying allocated interventions for a given trial participant (eg, drug dose change in response to harms, participant request, or improving/worsening disease) | 24-25 |
|  | 11c | Strategies to improve adherence to intervention protocols, and any procedures for monitoring adherence (eg, drug tablet return, laboratory tests) | 16 (Table 3) |
|  | 11d | Relevant concomitant care and interventions that are permitted or prohibited during the trial | 12-13 (Table 2) |
| Outcomes | 12 | Primary, secondary, and other outcomes, including the specific measurement variable (eg, systolic blood pressure), analysis metric (eg, change from baseline, final value, time to event), method of aggregation (eg, median, proportion), and time point for each outcome. Explanation of the clinical relevance of chosen efficacy and harm outcomes is strongly recommended | 16-17 (Table 4) |
| Participant timeline | 13 | Time schedule of enrolment, interventions (including any run-ins and washouts), assessments, and visits for participants. A schematic diagram is highly recommended (see Figure) | 11 (Figure 1) |
| Sample size | 14 | Estimated number of participants needed to achieve study objectives and how it was determined, including clinical and statistical assumptions supporting any sample size calculations | 25 |
| Recruitment | 15 | Strategies for achieving adequate participant enrolment to reach target sample size | 11-12 |
| **Methods: Assignment of interventions (for controlled trials)** | | |  |
| Allocation: |  |  |  |
| Sequence generation | 16a | Method of generating the allocation sequence (eg, computer-generated random numbers), and list of any factors for stratification. To reduce predictability of a random sequence, details of any planned restriction (eg, blocking) should be provided in a separate document that is unavailable to those who enrol participants or assign interventions | 14 |
| Allocation concealment mechanism | 16b | Mechanism of implementing the allocation sequence (eg, central telephone; sequentially numbered, opaque, sealed envelopes), describing any steps to conceal the sequence until interventions are assigned | 14 |
| Implementation | 16c | Who will generate the allocation sequence, who will enrol participants, and who will assign participants to interventions | 14 |
| Blinding (masking) | 17a | Who will be blinded after assignment to interventions (eg, trial participants, care providers, outcome assessors, data analysts), and how | 14 |
|  | 17b | If blinded, circumstances under which unblinding is permissible, and procedure for revealing a participant’s allocated intervention during the trial | 14 |
| **Methods: Data collection, management, and analysis** | | |  |
| Data collection methods | 18a | Plans for assessment and collection of outcome, baseline, and other trial data, including any related processes to promote data quality (eg, duplicate measurements, training of assessors) and a description of study instruments (eg, questionnaires, laboratory tests) along with their reliability and validity, if known. Reference to where data collection forms can be found, if not in the protocol | 17-18 |
|  | 18b | Plans to promote participant retention and complete follow-up, including list of any outcome data to be collected for participants who discontinue or deviate from intervention protocols | 17-18 |
| Data management | 19 | Plans for data entry, coding, security, and storage, including any related processes to promote data quality (eg, double data entry; range checks for data values). Reference to where details of data management procedures can be found, if not in the protocol | 23-24 |
| Statistical methods | 20a | Statistical methods for analysing primary and secondary outcomes. Reference to where other details of the statistical analysis plan can be found, if not in the protocol | 26 |
|  | 20b | Methods for any additional analyses (eg, subgroup and adjusted analyses) | 26 |
|  | 20c | Definition of analysis population relating to protocol non-adherence (eg, as randomised analysis), and any statistical methods to handle missing data (eg, multiple imputation) | 26 |
| **Methods: Monitoring** | | |  |
| Data monitoring | 21a | Composition of data monitoring committee (DMC); summary of its role and reporting structure; statement of whether it is independent from the sponsor and competing interests; and reference to where further details about its charter can be found, if not in the protocol. Alternatively, an explanation of why a DMC is not needed | Low-risk so DMC not needed. Trial advisory committee details in page 25 |
|  | 21b | Description of any interim analyses and stopping guidelines, including who will have access to these interim results and make the final decision to terminate the trial | N/A - low-risk intervention |
| Harms | 22 | Plans for collecting, assessing, reporting, and managing solicited and spontaneously reported adverse events and other unintended effects of trial interventions or trial conduct | 24-25 |
| Auditing | 23 | Frequency and procedures for auditing trial conduct, if any, and whether the process will be independent from investigators and the sponsor | Unplanned – will be conducted by ethics board(s) |
| **Ethics and dissemination** | | |  |
| Research ethics approval | 24 | Plans for seeking research ethics committee/institutional review board (REC/IRB) approval | 2 |
| Protocol amendments | 25 | Plans for communicating important protocol modifications (eg, changes to eligibility criteria, outcomes, analyses) to relevant parties (eg, investigators, REC/IRBs, trial participants, trial registries, journals, regulators) | 23 |
| Consent or assent | 26a | Who will obtain informed consent or assent from potential trial participants or authorised surrogates, and how (see Item 32) | 13-14 |
|  | 26b | Additional consent provisions for collection and use of participant data and biological specimens in ancillary studies, if applicable | 14 |
| Confidentiality | 27 | How personal information about potential and enrolled participants will be collected, shared, and maintained in order to protect confidentiality before, during, and after the trial | 23 |
| Declaration of interests | 28 | Financial and other competing interests for principal investigators for the overall trial and each study site | 2 |
| Access to data | 29 | Statement of who will have access to the final trial dataset, and disclosure of contractual agreements that limit such access for investigators | 27 |
| Ancillary and post-trial care | 30 | Provisions, if any, for ancillary and post-trial care, and for compensation to those who suffer harm from trial participation | No such provisions given low-risk of intervention |
| Dissemination policy | 31a | Plans for investigators and sponsor to communicate trial results to participants, healthcare professionals, the public, and other relevant groups (eg, via publication, reporting in results databases, or other data sharing arrangements), including any publication restrictions | 25 |
|  | 31b | Authorship eligibility guidelines and any intended use of professional writers | Journal authorship guidelines will be followed. No plans to use professional writers |
|  | 31c | Plans, if any, for granting public access to the full protocol, participant-level dataset, and statistical code | None |
| **Appendices** |  |  |  |
| Informed consent materials | 32 | Model consent form and other related documentation given to participants and authorised surrogates | PDFs in Supplementary Appendix |
| Biological specimens | 33 | Plans for collection, laboratory evaluation, and storage of biological specimens for genetic or molecular analysis in the current trial and for future use in ancillary studies, if applicable | None |

*It is strongly recommended that this checklist be read in conjunction with the SPIRIT 2013 Explanation & Elaboration for important clarification on the items. Amendments to the protocol should be tracked and dated. The SPIRIT checklist is copyrighted by the SPIRIT Group under the Creative Commons “[Attribution-NonCommercial-NoDerivs 3.0 Unported](http://www.creativecommons.org/licenses/by-nc-nd/3.0/)” license.

**S2 Appendix: *Completed World Health Organization Trial Registration Data Set***

| **Data category** | **Information** |
| --- | --- |
| Primary registry and trial identifying number | ClinicalTrials.gov NCT05235828 |
| Date of registration in primary registry | February 11th, 2022 |
| Secondary identifying numbers | Mount Sinai Hospital Research Ethics Board: MSH 21-0275-A |
| Source(s) of monetary or material support | Centre of Excellence on PTSD and Related Mental Health Conditions (now called “Atlas Institute for Veterans and Families”) |
| Primary sponsor | Mount Sinai Hospital (Lunenfeld-Tanenbaum Research Institute – Bridgepoint Campus) |
| Secondary sponsor(s) | Centre of Excellence on PTSD and Related Mental Health Conditions |
| Contact for public queries | *AS*, MD CCFP, abhimanyu.sud@utoronto.ca |
| Contact for scientific queries | Abhimanyu Sud, MD CCFP Department of Family and Community Medicine, Faculty of Medicine, University of Toronto; Institute of Health Policy, Management and Evaluation, Dalla Lana School of Public Health, University of Toronto |
| Public title | Sudarshan Kriya Yoga (SKY) for Canadian veterans with PTSD: a nation-wide effectiveness and implementation evaluation |
| Scientific title | Sudarshan Kriya Yoga (SKY) for Canadian veterans with PTSD: a nation-wide effectiveness and implementation evaluation |
| Countries of recruitment | Canada |
| Health condition(s) or problem(s) studied | Post-traumatic stress disorder (PTSD) |
| Intervention(s) | Intervention: *Sudarshan Kriya Yoga* |
| Key inclusion and exclusion criteria | Ages eligible for study: *>18 years* Sexes eligible for study: *both* Accepts healthy volunteers: *no*  Key inclusion criteria: *post-traumatic stress disorder (PCL-5 score ≥38); Canadian military or RCMP veteran; >18 years of age;*  Key exclusion criteria: *other major psychiatric condition including moderate to severe substance use disorder, psychosis, bipolar I, schizophrenia, cognitive impairment, uncontrolled, seizure disorder, and imminent risk of suicide; that would practicing a form of mind-body intervention.* |
| Study type | Hybrid type II (effectiveness and implementation study) Allocation: *randomized* Intervention model: waitlist control  Masking: *single-blind (outcomes assessor)* Primary purpose: *Effectiveness and implementation of intervention* |
| Date of first enrolment | May 2022 (anticipated) |
| Target sample size | 200 |
| Recruitment status | Recruiting |
| Primary outcome(s) | Change in PTSD symptomology measured on the PCL-5 Time frame: baseline, 6 weeks, 12 weeks, 30 weeks |
| Key secondary outcomes | Change in quality of life (SF-36), depressive (PHQ-9), pain (BPI), anxiety (GAD-7) symptoms  Time frame: baseline, 6 weeks, 12 weeks, 30 weeks |

**S3 Appendix: List of organizations and clinics supporting trial recruitment**

*Atlas Institute for Veterans and Families*

The Atlas Institute for Veterans and Families, formerly known as the Centre of Excellence (COE) on PTSD and Related Mental Health Conditions, is an organization funded by Veterans Affairs Canada (VAC) which is dedicated to supporting Veterans and their families through collective action.

*The Royal Canadian Legion*

The Royal Canadian Legion (‘the Legion’), is a non-profit organization that is dedicated to supporting military and RCMP veterans as well as their families. Members include current and former serving members of the Canadian Armed Forces and RCMP as well as their families.

*RCMP veterans’ Association (RCMPVA)*

The RCMPVA is a national organization dedicated to serving former members of the RCMP, especially with transitioning after leaving the force and with accessing benefits through VAC.

*Veterans Alliance of Canada*

The Veterans Alliance of Canada is a non-profit organization based in Winnipeg, Manitoba that assists veterans, public safety personnel (PSP), and civilians with access to alternative treatments for disorders such as PTSD, anxiety, and depression.

*Women Warriors Healing Garden (WWHG)*

WWHG is a volunteer-driven, non-profit organization based in Ontario. They run therapeutic programs for Canadian veterans in underrepresented groups, including women, visible minorities, indigenous peoples, and members of the 2SLGBTQ+ community.

*Prospect Human Services*

The Forces@Work program at Prospect Human Services supports members of the Canadian Armed Forces and veterans across Alberta in successfully transitioning to suitable civilian employment.

*Wounded Warriors Canada (WWC)*

WWC is a national mental health service provider that serves Canadian veterans, public safety personnel, and their families.

*Operational Stress Injury (OSI) Clinics*

OSI clinics provide essential treatment and support for Canadian military and RCMP veterans with service-related mental health issues. Several OSI clinics and their associated satellite clinics are collaborating to support recruitment efforts for this study. These clinics regularly receive new referrals from VAC including current members of the CAF or RCMP, veterans of the CAF or RCMP, and their family members. The following OSI clinics are supporting recruitment in this study:

● Nova Scotia Operational Stress Injury (OSI) Clinic in Dartmouth, NS

● Quebec Operational Stress Injury (OSI) Clinic in Quebec City, QC

● St. Joseph's Operational Stress Injury (OSI) Clinic in London, ON

*CHANGEpain Clinics*

CHANGEpain is a clinic in Vancouver, British Columbia that works with clients with chronic pain. Among these clients are a subsection of Canadian veterans who receive services for their chronic pain. Over 50% of their veteran patients struggle with PTSD.

*FIT For Work*

FIT For Work is an occupational medical and rehabilitation provider with an interdisciplinary team of professionals located in several locations across Newfoundland, Canada. Their clients include Canadian veterans.

**S4 Appendix – Semi-structured Interview Guides**

**S4 Appendix A– RCT Participant Interview Guide**

**Facilitator Script**

Thank you for agreeing to participate in today’s interview. My name is [insert name] and I am part of the research team for the *Sudarshan Kriya Yoga (SKY)* *for Canadian Veterans with PTSD* study. We are interested in learning about your thoughts about the virtually-delivered SKY program that you completed as part of this study. As noted in the consent form, you can skip questions or cease participating at any time without repercussions. The consent form also states that we will be audio-recording the interview. Is that okay? Do you have questions before we begin? [Facilitator turns on audio-recorder].

**Questions for those who completed the SKY program**

1. How did you find the virtually-delivered SKY (v-SKY) program? What were your expectations?
2. What worked well in the v-SKY program? What didn’t? What are your reasons?
3. What were the perceived benefits of the v-SKY program? What were the drawbacks?
   1. What was specifically helpful/unhelpful for your PTSD?
4. Was being in a group setting an advantage or disadvantage for you? Can you please explain your answer? What was it like being in a group of people specifically made up of other veterans with PTSD? What was it like being part of a group with other people who were more/less affected by the same mental health diagnosis?
5. How did you find the virtual (i.e. Zoom) environment for the class? What are your reasons for this?
6. Were there any barriers to your participation (in relation to attendance, completion of tasks/homework, technology, etc.?)
7. Was there anything that made it easier to participate (in relation to attendance, completion of tasks/homework, technology, etc.?)
8. What encouraged/discouraged you to complete homework tasks?
9. What are your thoughts on the duration of the course? Consider the total 6-week program, the 15 sessions, and the length of each session.
   1. How did you find the first week, which involved 5 sessions at 3-hours each?
   2. How about weeks 2-6, which involved 2 sessions per week at 1-hour each?
10. Have you noticed any differences in yourself (whether positive or negative) since completing the v-SKY program? If so, what?
    1. [Prompt about differences in terms of their PTSD symptoms]
11. Have people around you (spouse, family, friends, clinicians, etc.) noticed any differences (whether positive or negative) since completing the v-SKY program?
    1. [Prompt about differences in terms of their PTSD symptoms]
12. This program takes a holistic approach (Discussion, break out rooms, breathing techniques, grounding techniques, SKY, Yoga, journaling, mindfulness, etc), were there elements of the program that appealed or did not appeal to you?
13. How did you find the Ujjayi or “Victorious Breath” component?
    1. What worked/did not and why?
    2. How was the Ujjayi or “Victorious Breath” explained to you?
    3. Was the Ujjayi or “Victorious Breath” challenging for you? In what way?
14. How did you find the Bhastrika or “Bellows Breath,” component?
    1. What worked/did not and why?
    2. How was the Bhastrika or “Bellows Breath,” explained to you?
    3. Was the Bhastrika or “Bellows Breath,” challenging for you? In what way?
15. How did you find the “om” meditation chants component?
    1. What worked/did not and why?
    2. How was the “om” meditation chants component explained to you?
    3. Was the “om” meditation chants component challenging for you? In what way?
16. How did you find the sudarshan kriya (advanced cyclical breathing) component?
    1. What worked/did not and why?
    2. How was the sudarshan kriya (advanced cyclical breathing) explained to you?
    3. Was the sudarshan kriya (advanced cyclical breathing) challenging for you? In what way?
17. Do you plan to/are you currently practicing SKY techniques on a regular basis? If so, how often? If not, why?
18. Can you suggest improvements for future v-SKY programs in any of the areas we have covered/or not covered and you feel are important?
19. Would you recommend the v-SKY program to other veterans with PTSD? Why?
    1. Would you recommend it to anyone else? If so, who? What other groups of people do you think might benefit?
20. Since completing the v-SKY program, have you noticed any effects (positive or negative) on your sense of physical wellbeing? If so, what?
21. Since completing the v-SKY program, have you noticed any effects (positive or negative) on your sense of mental wellbeing? If so, what?

***Thank you so much for your time today and sharing your experience.***

**Questions for those who did NOT complete the SKY program**

1. Did you participate in any sessions for the virtually-delivered SKY (v-SKY) program? How many? What were your expectations?
   1. Why did you not finish/start the program?
2. What worked well in the v-SKY program? What didn’t? What are your reasons?
3. Was the v-SKY program helpful or not? In what way?
   1. What was specifically helpful/unhelpful for your PTSD?
4. Was being in a group an advantage or disadvantage for you? Can you please explain your answer? What was it like being in a group of people specifically made up of other veterans with PTSD? What was it like being part of a group with other people who were more/less affected by the same mental illness?
5. How did you find the virtual (i.e. Zoom) environment for the class? What are your reasons for this?
6. Were there any barriers to your participation (in relation to attendance, completion of tasks/homework, technology, etc.?)
   1. Did these barriers contribute to you not starting/finishing the v-SKY program?
7. Was there anything that would have made it easier to participate (in relation to attendance, completion of tasks/homework, technology, etc.?)
8. What are your thoughts about the homework tasks?
9. What are your thoughts on the duration of the course? Consider the total 6-week program, the 15 sessions, and the length of each session.
   1. What were your thoughts about the first week, which involved 5 sessions at 3-hours each?
   2. How about weeks 2-6, which involved 2 sessions per week at 1-hour each?
10. Did your randomization outcome (active or wait list control) play a role in your decision not to start/finish the v-SKY program?
11. Have people around you (spouse, family, friends, clinicians, etc.) noticed any differences (whether positive or negative) since joining this research study?
    1. [Prompt about differences in terms of their PTSD symptoms]
12. Do you have any suggestions for how we can make the v-SKY programs more accessible for you and people like yourself?
13. Can you suggest improvements for future v-SKY programs in any of the areas we have covered/or not covered and you feel are important?
14. Would you recommend the v-SKY program to other veterans with PTSD? Why?
    1. Would you recommend it to anyone else? If so, who? What other groups of people do you think might benefit?

***Thank you so much for your time today and sharing your experience.***

**S4 Appendix B – SKY Instructor Interview Guide**

**Facilitator Script**

Thank you for agreeing to participate in today’s interview. My name is [insert name] and I am part of the research team for the *Sudarshan Kriya Yoga (SKY)* *for Canadian Veterans with PTSD* study. We are interested in learning about your thoughts related to the implementation of virtually-delivered SKY (v-SKY). As noted in the consent form, you can skip questions or cease participating at any time without repercussions. The consent form also states that we will be audio-recording the interview. Is that okay? Do you have questions before we begin? [Facilitator turns on audio-recorder].

**Questions**

1. How did you find the virtually-delivery of the SKY program?
   1. How does it differ from the in-person program?
2. What are other differences for in-person vs virtually-delivered services? Do any of these differences change for v-SKY?
3. Were there any barriers to participation (in relation to attendance, completion of tasks/homework, technology, etc) for participants in the v-SKY program?
4. Was there anything that made it easier for participants in the v-SKY program to participate (in relation to attendance, completion of tasks/homework, technology, etc)?
5. Do you have any suggestions for how to make the v-SKY program more accessible for Canadian veterans?
6. Was instructing in a group setting an advantage or disadvantage for you? Can you please explain your answer?
7. What are the perceived benefits of the v-SKY program? What are the drawbacks?
   1. What do you believe is specifically helpful/unhelpful for veterans with PTSD?
8. What kinds of veterans with PTSD do you think would benefit from v-SKY?
   1. Do your views differ depending on whether SKY is offered virtually or in-person?
9. Would you recommend this program to other groups of people? If so, who? What other groups of people do you think might benefit?
10. What do you believe contributes to whether or not participants continue using techniques that they learned in the v-SKY program?
11. How do you think other people who work with veterans with PTSD view v-SKY as an intervention for veterans with PTSD?
12. How do you see SKY potentially fitting into the veteran health care system?
13. What do you think would need to be in place to provide a routine v-SKY program for veterans with PTSD?
14. Do you think v-SKY for veterans with PTSD is something that VAC could/would support? Why/Why not?
15. What would the role of the IAHV and AOLF be if the v-SKY program were to be sustained in the health care program? What about the role of individual SKY instructors?
16. How would you assess the overall worth of having v-SKY for veterans with PTSD?
17. How would you judge if v-SKY for veterans with PTSD was a good thing or not in the long term?
18. Is there anything else you would like to share?

***Thank you so much for your time today and sharing your experience.***

**S4 Appendix C – Health Professionals and Administrators Interview Guide**

**Facilitator Script**

Thank you for agreeing to participate in today’s interview. My name is [insert name] and I am part of the research team for the *Sudarshan Kriya Yoga (SKY)* *for Canadian Veterans with PTSD* study. We are interested in learning about your thoughts related to the implementation of virtually-delivered SKY (v-SKY). As noted in the consent form, you can skip questions or cease participating at any time without repercussions. The consent form also states that we will be audio-recording the interview. Is that okay? Do you have questions before we begin? [Facilitator turns on audio-recorder].

**Questions**

1. What are your views on v-SKY?
   1. Can you share your thoughts regarding if/how v-SKY differs from other psychological treatments?
2. Could you describe your views on the purpose of v-SKY?
3. Do you think having SKY available for veterans with PTSD would assist in your work? If so, why? If not, why?
   1. Do your views differ depending on whether SKY is offered virtually or in-person?
4. How do you think other people who work with veterans with PTSD view v-SKY as a treatment for veterans with PTSD?
5. What kinds of veterans with PTSD do you think would benefit from v-SKY?
   1. Do your views differ depending on whether SKY is offered virtually or in-person?
6. Is v-SKY something you would consider referring veteran clients/patients for? If yes, how would you go about this? If not, what issues would make you unwilling to refer?
7. Have you noticed any effects of the SKY intervention on the patients/clients that you work with? Please elaborate.
8. Have you noticed any effects of the SKY intervention on the overall clinical/organizational environment? Please elaborate.
9. Would you be interested in learning more about v-SKY? Would a continuing professional development event be of interest? If yes, why? If not, why?
10. What information would you require in order to refer a client/patient for a v-SKY?
    1. What would you want to know about the intervention in order to make a referral?
    2. Do your views differ depending on whether SKY is offered virtually or in-person?
11. What do you think would need to be in place to provide a routine v-SKY program for veterans with PTSD?
12. Do you think v-SKY for veterans with PTSD is something that VAC could/would support? Why/Why not?
13. What sort of feedback would you want from those running/attending a v-SKY program for veterans with PTSD?
14. How would you assess the overall worth of having v-SKY for veterans with PTSD?
15. How would you assess whether v-SKY had been worthwhile for your patient/client?
16. How would you judge if v-SKY for veterans with PTSD was a good thing or not in the long term? What criteria might stop you referring?
17. What are some notable differences between in-person vs virtually-delivered services? Do any of these differences change for v-SKY?
18. Is there anything else you would like to share?

***Thank you so much for your time today and sharing your experience.***
